# Behavior of bacteriome symbionts during transovarial transmission and development of the Asian citrus psyllid

**DOI:** 10.1371/journal.pone.0189779

**Published:** 2017-12-14

**Authors:** Hiroki Dan, Naoya Ikeda, Masaya Fujikami, Atsushi Nakabachi

**Affiliations:** 1 Department of Environmental and Life Sciences, Toyohashi University of Technology, Toyohashi, Aichi, Japan; 2 Electronics-Inspired Interdisciplinary Research Institute (EIIRIS), Toyohashi University of Technology, Toyohashi, Aichi, Japan; United States Department of Agriculture, UNITED STATES

## Abstract

The Asian citrus psyllid *Diaphorina citri* Kuwayama (Hemiptera: Liviidae) is a serious pest worldwide, transmitting *Candidatus* Liberibacter spp. (*Alphaproteobacteria*), the causative agents of a devastating citrus disease known as huanglongbing or greening disease. In a symbiotic organ called the bacteriome, *D*. *citri* possesses an organelle-like defensive symbiont, *Candidatus* Profftella armatura (*Betaproteobacteria*), and a nutritional symbiont, *Ca*. Carsonella ruddii (*Gammaproteobacteria*). Drastically reduced symbiont genomes and metabolic complementarity among the symbionts and *D*. *citri* indicate their mutually indispensable association. Moreover, horizontal gene transfer between the *Profftella* and *Liberibacter* lineages suggests ecological and evolutionary interactions between the bacteriome symbiont and the HLB pathogen. Using fluorescence *in situ* hybridization, we examined the behavior of *Profftella* and *Carsonella* during transovarial transmission and the development of *D*. *citri*. In the bacteriomes of sexually-mature female adults, symbionts transformed from an extremely elongated tubular form into spherical or short-rod forms, which migrated toward the ovary. The symbionts then formed mosaic masses, which entered at the posterior pole of the vitellogenic oocytes. After anatrepsis, *Carsonella* and *Profftella* migrated to the central and peripheral parts of the mass, respectively. Following the appearance of host nuclei, the mass cellularized, segregating *Carsonella* and *Profftella* in the central syncytium and peripheral uninucleate bacteriocytes, respectively. Subsequently, the uninucleate bacteriocytes harboring *Profftella* assembled at the posterior pole, while the syncytium, containing *Carsonella*, sat on the anterior side facing the germ band initiating katatrepsis. During dorsal closure, the syncytium was divided into uninuclear bacteriocytes, which surrounded the mass of bacteriocytes containing *Profftella*. Once fully surrounded, the bacteriocyte mass containing *Profftella* was fused into a syncytium. Prior to hatching, a pair of wing-like protrusions arose from both lateral sides of the bacteriome, which continued to grow throughout the nymphal stages. These findings provide a foundation for better understanding the intricate relationship between *D*. *citri* and its microbiota.

## Introduction

The Asian citrus psyllid *Diaphorina citri* Kuwayama (Hemiptera: Sternorrhyncha: Psylloidea: Liviidae) is an important agricultural pest that transmits *Candidatus* Liberibacter spp. (*Alphaproteobacteria*), the causative agents of a devastating citrus disease known as huanglongbing (HLB) or greening disease. All commercial citrus cultivars are susceptible to HLB, and a long latent period following infection allows rapid spread of the disease worldwide. Because HLB is currently incurable, controlling the *D*. *citri* vector is presently the most crucial part of HLB management [1].

*D*. *citri* possesses a symbiotic organ called the bacteriome in its hemocoel. The bacteriome harbors two distinct species of vertically transmitted symbionts: *Candidatus* Carsonella ruddii (*Gammaproteobacteria*) and *Ca*. Profftella armatura (*Betaproteobacteria*) [2]. *Carsonella* is a typical nutritional symbiont, providing its host with essential amino acids that are scarce in the diet of phloem sap. In contrast, *Profftella* appears to be an organelle-like defensive symbiont, producing toxins that protect the host from natural enemies. A mutually indispensable tripartite association among *D*. *citri* and the two symbionts is strongly suggested by the drastically reduced symbiont genomes, and by the metabolic complementarity among the organisms [2]. These features are partly the result of horizontal gene transfer between partners [3,4]. Notably, the *Liberibacter* lineage has also horizontally acquired a gene from the *Profftella* lineage, demonstrating ecological and evolutionary interactions between the HLB pathogen and the bacteriome symbiont [5]. Thus, revealing the behavior of *Carsonella* and *Profftella* during the host life cycle is essential for understanding the biology of *D*. *citri* and its associated microbiota, which would aid in the development of efficient means to control HLB.

As early as in 1937, Profft described the dynamics of apparently varied symbionts of several psyllid species using classical staining methods ([6], reviewed in [7]). However, these methods can neither identify nor even distinguish symbiont species distinctly. Furthermore, these early papers included only a limited number of figures, all of which were hand drawn, and lacked information on *D*. *citri*. Therefore, in the present study, we analyzed the transovarial transmission and dynamics of *Carsonella* and *Profftella* during the embryonic and postembryonic development of *D*. *citri* using fluorescence *in situ* hybridization (FISH).

## Materials and methods

### Psyllids

An established colony of *D*. *citri*, originally collected from Amami Oshima Island, Kagoshima, Japan, was maintained on *Murraya paniculata* Jack (Rutaceae) with a 16-h light period (28°C) and 8-h dark period (23°C). For egg collection, adult females were allowed to mate and oviposit on *Citrus junos* Tanaka (Rutaceae) seedlings maintained under the same conditions as described above.

### Fixation and decolorization

Insect materials for FISH analysis were prepared as reported previously [8], with some modifications. Embryos, 1^st^ to 5^th^ instar nymphs, and adults were fixed in Carnoy’s solution (ethanol:chloroform:glacial acetic acid, 6:3:1) at room temperature overnight. After washing with 100% ethanol, the fixed samples were treated with 6% H_2_O_2_ in 80% ethanol until sufficiently decolorized. The bleached samples were then washed with 100% ethanol.

### Tissue sectioning

Samples were infiltrated and embedded in polyester wax (VWR) [9], and then sliced into serial sections (5 μm thickness) using a rotary microtome RV-240 (Yamato Koki). The sections were mounted on silane-coated Platinum Pro glass slides (Matsunami Glass), and dewaxed in 100% ethanol. The samples were then rehydrated using a graded ethanol to phosphate-buffered saline (PBS) series in descending concentrations.

### *In situ* hybridization of tissue sections

Probes Car1 (5′-CGCGACATAGCTGGATCAAG-3′) [10] and SSDC_127247 (5′-GACCCTCTGTATGCACCATT-3′) [2] were used to specifically detect 16S rRNA from *Carsonella* and *Profftella*, respectively. Car1 and SSDC_127247 were 5′-labeled with Alexa Fluor 594 and Alexa Fluor 488, respectively. The tissue sections on glass slides were pre-incubated with hybridization buffer [20 mM Tris-HCl (pH8.0), 0.9M NaCl, 0.01% sodium dodecyl sulfate, 20% formamide], without the probe, at room temperature for 1 h. The sections were then incubated at room temperature overnight with hybridization buffer containing 100 nM of each of the probes. The samples were then washed twice with PBS, and mounted in ProLong Gold antifade reagent with DAPI (Thermo Fisher Scientific) using a cover slip. The slides were examined by fluorescence microscopy (BX-53; Olympus) or confocal laser microscopy (A1; Nikon).

### *In situ* hybridization of whole mount samples

The decolorized and washed samples were hydrated with PBSTx (0.8% NaCl, 0.02% KCl, 0.115% Na_2_HPO_4_, 0.02% KH_2_PO_4_, 0.3% Triton X-100), pre-incubated three times (20 min per incubation) with the hybridization buffer minus the probe, and then incubated with the hybridization buffer containing 100 nM of each of the probes at room temperature overnight. After washing twice with PBSTx, the samples were transferred onto glass slides with spacers, and mounted in ProLong Gold antifade reagent with DAPI using a cover slip. The specimens were examined using a Nikon A1 laser scanning confocal microscope, and acquired images were analyzed using NIS-elements AR Analysis 4.10 software (Nikon).

## Results and discussion

### Migration of symbionts from the bacteriome to the ovary

**Fig 1:**
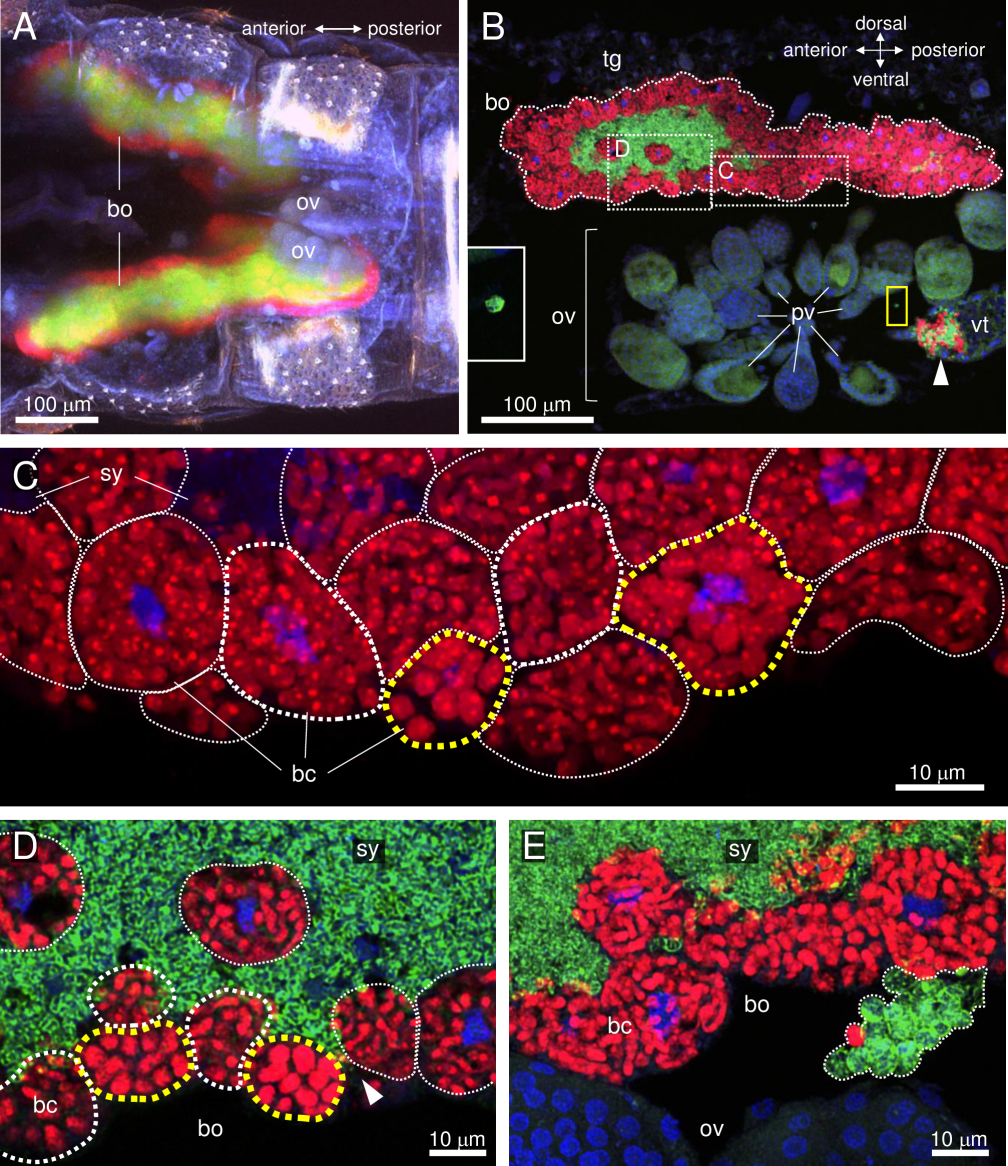
Confocal micrographs of FISH carried out in the abdomen of an adult female *D*. *citri*. Red (Alexa Fluor 594), green (Alexa Fluor 488), and blue (DAPI) signals indicate *Carsonella*, *Profftella*, and the host nuclei, respectively. (A) Whole-mount FISH image [maximum intensity projection (MIP)] of the ventral view of the abdomen of an adult female on the day of eclosion showing the bacteriome and immature ovaries. (B) FISH image (MIP) of a sagittal cross-section of the abdomen of an adult female at 5 days post-eclosion. *Carsonella* can be seen within the uninucleate bacteriocytes on the surface of the bacteriome, while *Profftella* is encased in syncytial cytoplasm at the center of the bacteriome. *Carsonella* and *Profftella* signals can also be seen in an oocyte at the vitellogenic stage (arrowhead), which is located in the ovariole constituting the ovary. Inset on the left is an enlarged image of the area in the yellow rectangle showing a *Profftella* cell in the hemocoel. (C) Enlarged image (MIP) of the bacteriome in the dotted rectangle of B, from which the Alexa Fluor 488 (*Profftella*) signals have been removed. Yellow dotted lines surround bacteriocytes containing large spherical *Carsonella* cells, while thin white dotted lines indicate bacteriocytes containing elongated thin tubular *Carsonella* cells. Bacteriocytes circled by thick white dotted lines contain *Carsonella* cells that appear to be in the process of transformation. (D) Enlarged image (optical section) of the area shown in B. Dotted lines indicate the same structures as described in C. The arrowhead indicates the syncytium harboring *Profftella*, which reaches the surface of the bacteriome. (E) FISH image (MIP) of another sagittal cross-section of the abdomen of the same individual shown in A–D. A mass of spherical *Carsonella* and *Profftella* cells exiting from the bacteriome is indicated within the dotted line. Abbreviations: bc, bacteriocyte; bo, bacteriome; ov, ovary; pv, previtellogenic oocyte; sy, syncytium; tg, tergite; vt, vitellogenic oocyte.

Translocation of *Carsonella* and *Profftella* from the bacteriome to the ovary was analyzed using female adults at 0, 3, 5, and 10 days post-eclosion. In the bacteriome, as reported previously [2], *Carsonella* was located in uninucleate bacteriocytes [11] on the surface of the organ, while *Profftella* was observed in the syncytial cytoplasm, located at the center of the organ (Fig 1A and 1B). Both *Carsonella* and *Profftella* are pleiomorphic, but are generally tubular in shape [2]. The ovaries of *D*. *citri* consist of nearly 50 ovarioles arranged in a bouquet, and are located ventrolaterally in the abdomen, just below the bacteriome [12] (Fig 1A and 1B). In adults at 0 and 3 days post-emergence, in which ovaries were small and all oocytes were at the previtellogenic stage, symbionts were observed only in the bacteriome, showing no sign of transmission to the ovary (Fig 1A).

At 5 and 10 days post-eclosion, spherical cells of both symbionts were observed in the ovary (Fig 1B) and the hemolymph (Fig 1B and 1E, S1 Movie), in addition to the tubular *Carsonella* and *Profftella* cells in the bacteriome. These cells were presumptively in the process of transovarial transmission and migration from the bacteriome to the ovary, respectively. The ovarioles in these adults contained both previtellogenic and vitellogenic oocytes [12]. As the symbionts were observed only in the latter with accumulated yolk, it is likely that symbiont infection occurs only at the vitellogenic stage (Fig 1B). The symbionts in the hemocoel are no longer sequestered within the host cells, enhancing their opportunity to interact with other microbes, including *Liberibacter* spp., which could potentially facilitate horizontal gene transfer among the bacteria [5]. In adults at 5 and 10 days post-eclosion, spherical *Carsonella* cells were also observed in several bacteriocytes facing the ovary, while the majority of bacteriocytes contained ordinary tubular *Carsonella* cells (Fig 1C, S2 Movie). This appears to reflect the transformation of *Carsonella* from tubular to spherical form to facilitate migration and infection into the oocyte. The mechanism of the transformation, which likely involves thickening and transverse cleavage, is yet to be elucidated, but the *D*. *citri* host must control the process as neither *Carsonella* nor *Profftella* possesses genes for cell division [2]. At the same stage (5 and 10 days post-eclosion) in the bacteriome, parts of the syncytium harboring *Profftella* had arrived at the surface of the organ (Fig 1D), which would facilitate the exit of *Profftella* from the bacteriome before migration toward the ovary (Fig 1E, S1 Movie).

### Infection of oocytes by symbionts

**Fig 2:**
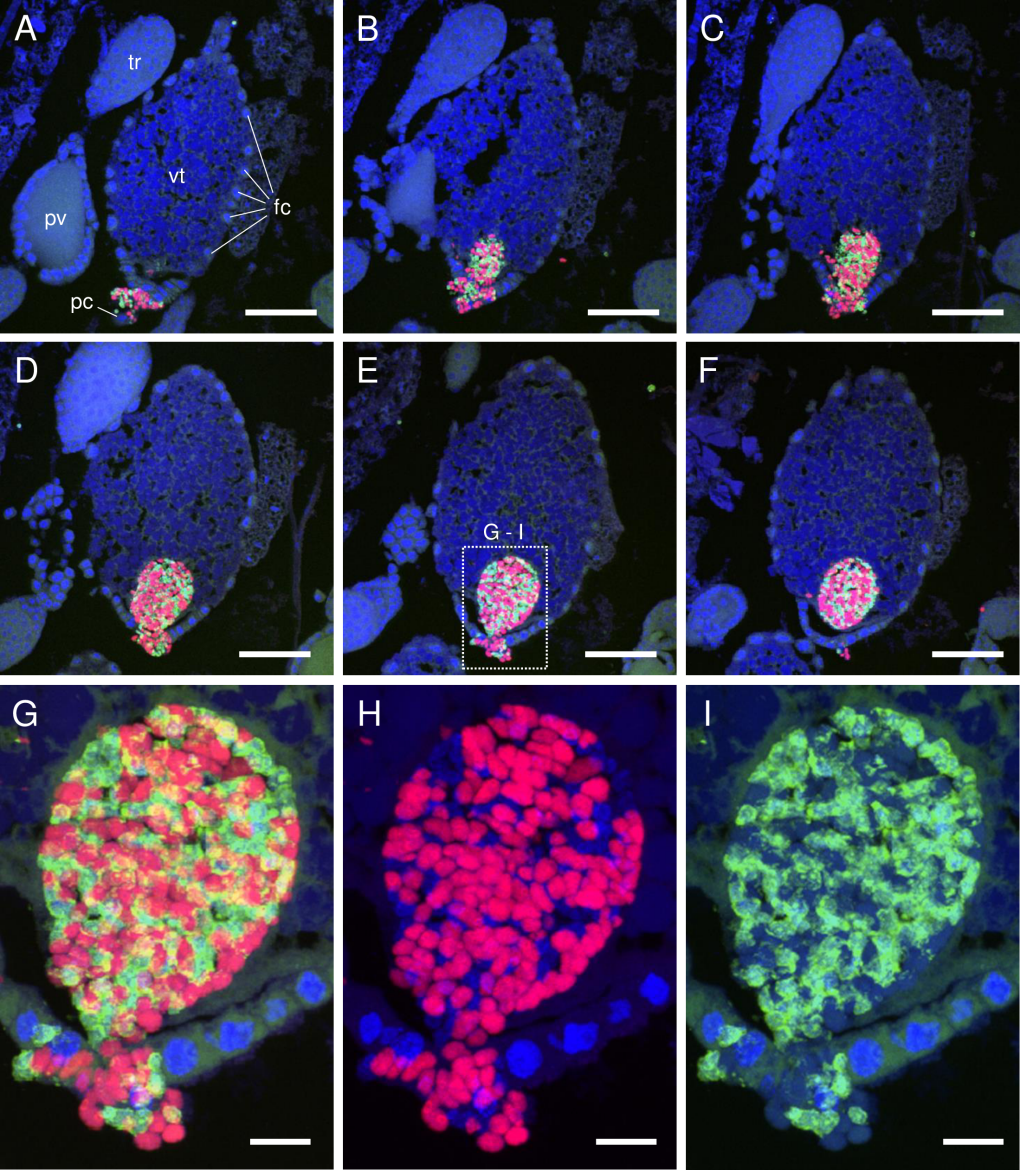
Confocal micrographs (MIP) showing transovarial transmission of *Carsonella* and *Profftella*. (A–F) FISH images showing an oocyte accepting symbionts in serial sections (5 μm thick) of an adult female *D*. *citri* at 5 days post-eclosion. Red (Alexa Fluor 594), green (Alexa Fluor 488), and blue (DAPI) signals indicate *Carsonella*, *Profftella*, and the host nuclei, respectively, unless otherwise stated. A mass of *Carsonella* and *Profftella* cells can be seen entering the posterior of a vitellogenic oocyte through follicle cells and the pedicel of the ovariole. (G) Enlarged image of the dotted rectangle shown in E. (H) Duplicate of image shown in G following the removal of Alexa Fluor 488 (*Profftella*) signals. Spherical or short rod-shaped *Carsonella* cells can be seen entering the posterior of the oocyte through follicle cells. Note that the shape of the *Carsonella* cells does not change after entering the oocyte. Some of the weak DAPI signals in this micrograph correspond to *Profftella* (see also I). (I) Duplicate of the image shown in G following the removal of Alexa Fluor 594 (*Carsonella*) signals. Spherical *Profftella* cells can be seen entering the posterior of the oocyte through follicle cells. Note that *Profftella* cells in the oocyte have already transformed from spherical to tubular form. Some of the weak DAPI signals in this micrograph correspond to *Carsonella*. Abbreviations: fc, follicle cell; pc, pedicel; pv, previtellogenic oocyte; tr, trophocyte; vt, vitellogenic oocyte. Scale bars: 50 μm in A–F, 10 μm in G–I.

Based on the observations described above, transmission of *Carsonella* and *Profftella* into *D*. *citri* eggs was examined using female adults at 5 days post-eclosion. Inspection of serial sections of oocytes accepting symbionts showed that a mass of *Carsonella* and *Profftella* cells entered at the posterior pole of the vitellogenic oocyte through follicle cells and the pedicel (ovariolar stalk) of the ovariole (Fig 2A–2I). This type of symbiont mass entering an oocyte is often referred to as “symbiont ball”, and appears to be a common feature of transovarial transmission of various intracellular microbes, including bacteria and even yeast-like fungal symbionts, in multiple insect lineages [7,13–18]. Interestingly, the spherical or short-rod shape of *Carsonella* did not change when entering the oocyte (Fig 2H, S3 Movie), whereas spherical *Profftella* cells transformed into a tubular form just after entering the oocyte (Fig 2I, S4 Movie). The shape of the symbionts is generally believed to reflect the duration of the intracellular symbiosis [7,19]. Buchner stated that “those (symbionts) of the syncytia, which often retain the original bacterial form, are the late-comers and that only some of them assume those bizarre growth forms as a result of long exposure to intracellular living” [7]. *Carsonella* is the primary symbiont, residing in all psyllid species reported to date [2,3,20,21], while *Profftella*, which is found only in *D*. *citri* so far, is the secondary symbiont and is therefore the late comer [2]. Nevertheless, *Profftella* reverted from the spherical infectious form to the “bizarre growth form” earlier than the older resident, *Carsonella*.

### Dynamics of the symbionts during embryogenesis

**Fig 3:**
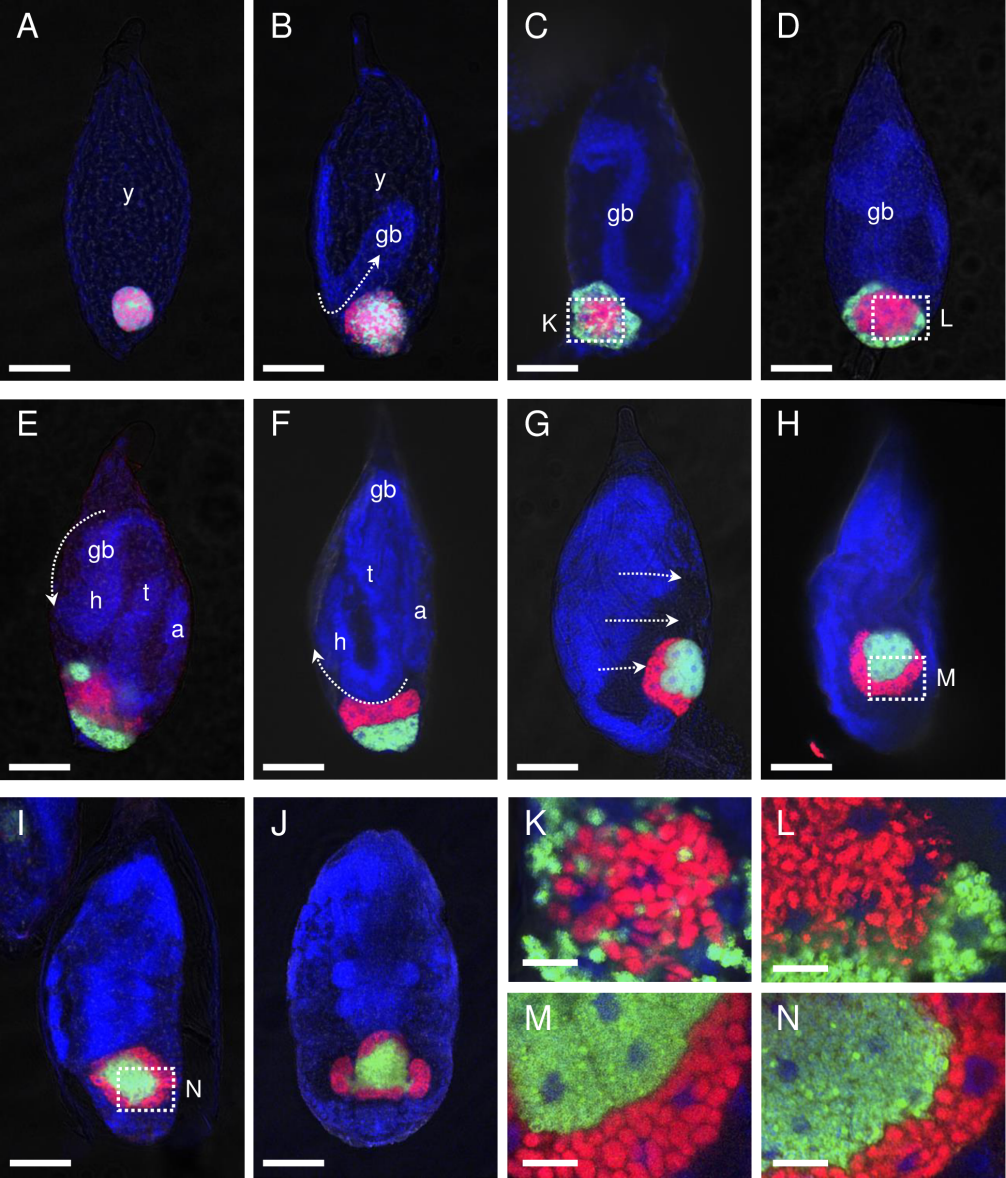
Confocal micrographs (optical sections) showing dynamics of *Carsonella* and *Profftella* during the embryogenesis of *D*. *citri*. Whole-mount FISH images of embryos. Red (Alexa Fluor 594), green (Alexa Fluor 488), and blue (DAPI) signals indicate *Carsonella*, *Profftella*, and the host nuclei, respectively. (A) Blastula-stage embryo collected at 0–6 h post-oviposition. A tightly aggregated ball-like mosaic mass of *Carsonella* and *Profftella* can be observed at the posterior pole of the embryo. (B) An embryo at 6–12 h post-oviposition. The symbiont mass was loosening. (C) An embryo at 12–18 h post-oviposition. *Carsonella* and *Profftella* can be observed in the central and peripheral parts of the mass, respectively. Host nuclei are observed in the symbiont mass. (D) An embryo18–24 h post-oviposition. *Carsonella* and *Profftella* can be observed in the central syncytium and peripheral uninucleate bacteriocytes, respectively. (E) An embryo at 24–30 h post-oviposition. The head of the germ band nears the proto-bacteriome. Uninucleate bacteriocytes harboring *Profftella* migrate toward the posterior pole. (F) A katatrepsis-stage embryo at 30–36 h post-oviposition. The uninucleate bacteriocytes harboring *Profftella* assemble at the posterior pole, whereas the syncytium containing *Carsonella* resides on the anterior side of the mass. (G) An embryo (36–48 h post-oviposition) in the process of dorsal closure. The syncytium harboring *Carsonella* is divided into uninuclear bacteriocytes and begins to surround the mass of *Profftella*-containing bacteriocytes. (H) An embryo at 48–60 h post-oviposition. The proto-bacteriome is settled within the embryo. (I) An embryo at 60–72 h post-oviposition. The mass of bacteriocytes harboring *Profftella* is fused into a syncytium, which is completely surrounded by uninuclear bacteriocytes containing *Carsonella*. (J) An embryo at 72–84 h post-oviposition. Two wing-like protrusions start to grow from the lateral sides of the bacteriome. (K) Enlarged image of the symbiont mass with host nuclei shown in C. (L) Enlarged image of the cellularized proto-bacteriome shown in D. (M) Enlarged image of the proto-bacteriome shown in H. *Profftella*-containing bacteriocytes are still uninucleate. (N) Enlarged image of the proto-bacteriome shown in I. *Profftella*-containing bacteriocytes are fused into a syncytium. Abbreviations: a, abdomen; gb, germ band; h, head; t, thorax; y, yolk. Scale bars: 50 μm for A–J, 10 μm for K–N. Arrows indicate the presumed direction of motion of the embryo.

**Fig 4:**
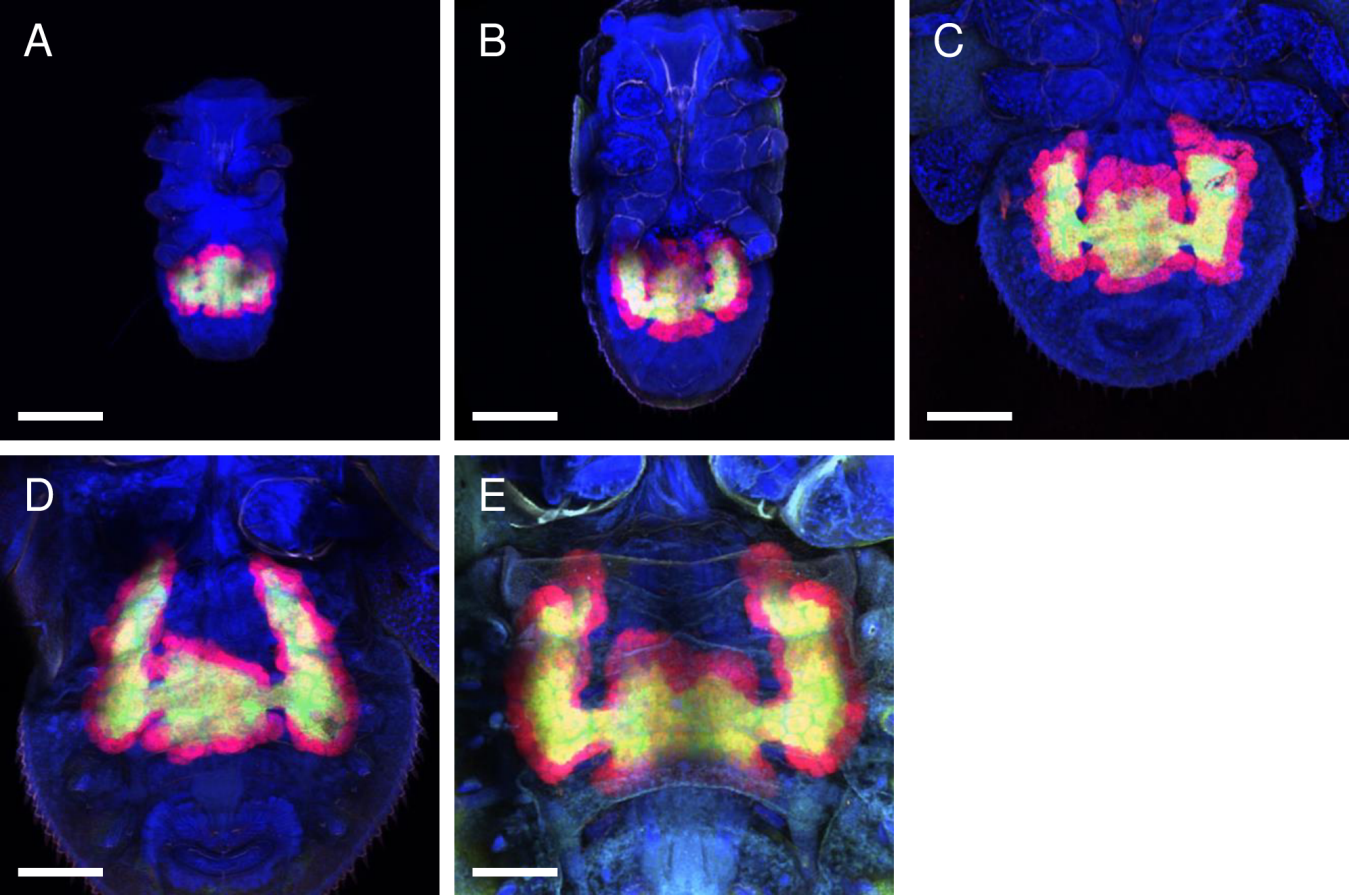
Confocal micrographs (MIP) of whole-mount FISH assays showing the bacteriomes of the *D*. *citri* nymphs. Red (Alexa Fluor 594), green (Alexa Fluor 488), and blue (DAPI) signals indicate *Carsonella*, *Profftella*, and the host nuclei, respectively. Bar: 100 μm. (A) 1^st^ instar. (B) 2^nd^ instar. (C) 3^rd^ instar. (D) 4^th^ instar. (E) 5^th^ instar. The bacteriome continuously increased in size and volume during nymphal development in proportion to the increase in body size. The pair of wing-like protrusions that emerged at the late embryonic stage continued to grow throughout the nymphal stages.

Formation of the bacteriome and dynamics of the symbionts during embryogenesis were analyzed using eggs collected every 6 or 12 h from plants where adult females were allowed to mate and oviposit. In blastula-stage embryos collected 0–6 h post- oviposition, a tightly aggregated ball-like mosaic mass (“symbiont ball”) of *Carsonella* and *Profftella* was observed at the posterior pole (Fig 3A). In embryos observed at 6–12 h post-oviposition, in which the newly formed germ band was at the anatrepsis stage (invagination into the central yolk) [22], the symbiont mass was loosening (Fig 3B). At 12–18 h post-oviposition, when the germ band extended, *Carsonella* and *Profftella* migrated to the central and peripheral parts of the mass, respectively. *Carsonella* cells started to elongate, and host nuclei appeared in the symbiont mass (Fig 3C and 3K). In embryos examined at 18–24 h post-oviposition, the proto-bacteriome appeared cellularized, with *Carsonella* and *Profftella* encased in the central syncytium and peripheral uninucleate bacteriocytes, respectively (Fig 3D and 3L). Notably, this arrangement is the opposite of that observed in the postembryonic bacteriome, in which *Carsonella* and *Profftella* are located in the peripheral uninuclear bacteriocytes and the central syncytium, respectively (Figs 1 and 4).

At the next stage of development (24–30 h post-oviposition), in which the head of the germ band was reaching the proto-bacteriome, uninucleate bacteriocytes harboring *Profftella* migrated toward the posterior pole (Fig 3E). In the subsequent katatrepsis stage (observed at 30–36 h post-oviposition), during which the embryo exits from the yolk [22], uninucleate bacteriocytes harboring *Profftella* assembled at the posterior pole, whereas the syncytium containing *Carsonella* was located on the anterior side of the mass (Fig 3F). At 36–48 h post-oviposition, embryos were in the process of dorsal closure, whereby the dorsal gap is closed by the fusion of epithelial cell sheets [22]. During this stage, the proto-bacteriome was located at the tip of the abdomen of the embryo. The syncytium, harboring *Carsonella*, was divided into uninuclear bacteriocytes, and was starting to surround the mass of bacteriocytes containing *Profftella* (Fig 3G). When the dorsal closure was completed (48–60 h post-oviposition), the proto-bacteriome was settled within the embryo, and the mass of bacteriocytes harboring *Profftella* was further surrounded by the uninuclear bacteriocytes containing *Carsonella* (Fig 3H and 3M). When the mass of bacteriocytes harboring *Profftella* was completely surrounded by the *Carsonella*-containing bacteriocytes (60–72 h post-oviposition), the former cells were fused into a syncytium (Fig 3I and 3N). At this point, the arrangement of the symbionts was the same as that seen in the postembryonic bacteriome, with *Carsonella* and *Profftella* in the peripheral uninuclear bacteriocytes and the central syncytium, respectively. At 72–84 h post-oviposition, two wing-like protrusions began to develop from both lateral sides of the bacteriome within the embryo (Fig 3J). The embryos then hatched at 84–96 h post-oviposition.

Profft studied the development of the bacteriome within *Psylla alni* (Psyllidae) [6], another psyllid species belonging to a different family from *D*. *citri* (Liviidae) [23]. Although few figures were presented regarding this subject, his descriptions suggest that the general trend in the formation of the bacteriome and the dynamics of symbionts, which are unidentified in *P*. *alni*, during embryogenesis is similar between the two psyllid species [6]. Whereas the mechanism of manipulating symbionts is generally unknown for insect endosymbiotic systems, the host actin was hypothesized to be involved in the process in several other previously studied insect lineages [24,25].

### Postembryonic development of the bacteriome

The life cycle of *D*. *citri* includes five nymphal instars [1], each of which were examined in the current study using the whole-mount FISH method. In accordance with the increase in body size, the bacteriome continuously increased in size and volume during the nymphal development (Fig 4A–4E). In addition, the pair of wing-like protrusions that arose in the late embryonic stage (Fig 3J) continued to grow throughout the nymphal stages (Fig 4A–4E). It appears that the shape of the bacteriome changes most dramatically during the adult eclosion, when the wing-like structures elongated further and became more eminent even than the original structure, which itself became a narrow central bridge connecting the two protrusions (Fig 1A).

## Supporting information

S1 Movie Three-dimensional reconstruction of the area shown in Fig 1E. (MP4)

S2 Movie Three-dimensional reconstruction of the area shown in Fig 1C. (MP4)

S3 Movie Three-dimensional reconstruction of the area shown in Fig 2H. (MP4)

S4 Movie Three-dimensional reconstruction of the area shown in Fig 2I. (MP4)
